# Whole-Genome Shotgun Sequences of Three Multidrug-Resistant *Mycobacterium tuberculosis* Strains Isolated from Morocco

**DOI:** 10.1128/genomeA.01275-17

**Published:** 2017-11-16

**Authors:** Lamiaa Lahlou, Nadia El Mrimar, Meriem Laamarti, Tarek Alouane, Mohammed A. Bendahou, Fatna Bssaibis, Yassine Ben Lahlou, Adil Zegmout, N. El Hafidi, Rachid ElJaoudi, Mohammed Frikh, Abdelhay Lemnouar, Mostafa Elouennass, Azeddine Ibrahimi

**Affiliations:** a Biotechnology Lab (MedBiotech Center), Rabat Medical and Pharmacy School, Mohammed V University, Rabat, Morocco; b Service de Bactériologie, Hôpital Militaire d’Instruction Mohammed V Equipe d’Épidémiologie et Résistance Bactérienne (ERB), Rabat Medical and Pharmacy School, Mohammed V University, Rabat, Morocco; c Service de Pneumologie, Hôpital Militaire d’Instruction Mohammed V Rabat Medical and Pharmacy School, Mohammed V University, Rabat, Morocco

## Abstract

Tuberculosis is a contagious disease that usually attacks the lungs but sometimes attacks other parts of the body, such as the kidneys, glands, and bones. It is an endemic and major public health problem in Morocco. Tuberculosis is transmitted through the airways via the inhalation of microdroplets containing *Mycobacterium tuberculosis*. We present here the whole-genome shotgun sequences of three multidrug-resistant *M. tuberculosis* strains isolated from Morocco.

## GENOME ANNOUNCEMENT

In cases of exposure to tuberculosis, 70% of individuals are not infected, while 30% develop an infection. Of infected individuals, 90% have a latent infection, while the remaining 10% develop tuberculosis (1, 2). Multidrug-resistant tuberculosis (MDR-TB) is caused by a TB bacterium that is resistant to at least isoniazid and rifampin, the two most potent drugs used to treat TB infection. Extensively drug-resistant TB (XDR-TB) is a form of MDR-TB with additional resistance to more anti-TB drugs that responds to fewer available drugs, such as fluoroquinolone and ethionamide. These drugs are used to treat all people with tuberculosis (3).

Here, we report the whole-genome shotgun sequences of three clinical strains of *Mycobacterium tuberculosis*, MTB2_M, MTB1_M, and MTB20_M, which were isolated from sputum specimens from three male patients and identified in the Department of Bacteriology, Mohammed V Rabat Military Training Hospital in Morocco. 

All strains (MTB2_M, MTB1_M, and MTB20_M) were cultured in Lowenstein-Jensen medium, and total genomic DNA was extracted using a GenoLyse kit (Hain Lifescience). DNA concentrations were determined using the NanoVuePlus spectrophotometer (Biochrom), and 1 ng of DNA was used to sequence the whole genome of the strain. Shotgun libraries were prepared from the extracted genomic DNA following the Nextera XT (V3) protocol (Illumina), and the Illumina MiSeq platform was used for the small-genome sequencing.

The genome sequence of each *M. tuberculosis* strain was determined by high-throughput sequencing performed on the Illumina MiSeq platform with a paired-end 250-bp sequencing kit. *De novo* assemblies were performed using SPAdes version 3.10 (4).

We obtained 1,092,302 reads with an average read length of 250 bp and approximately 41-fold coverage. The assembled reads generated a genome containing a total of 4,111,412 bp for MTB2_M. For MTB20_M the assembled reads generated a genome containing a total of 4,358,959 bp with an *N*_50_ value of 28,431 bp, and for MTB1_M the assembled reads generated a genome containing a total of 4,064,236 bp with an *N*_50_ value of 20,340 bp.

The three genomes were annotated using the Rapid Annotations using Subsystems Technology (RAST) server (5, 6) to predict subsystems, and Prokka was used to predict RNAs and coding sequences (7).

All genome sequences have a mean GC content of 65.3%. MTB2_M contains 379 subsystems with 96 virulence, disease, and defense subsystems; 4,116 protein-coding genes; and 47 RNAs, while MTB1_M contains 292 subsystems with 65 virulence, disease, and defense subsystems; and 3,196 protein-coding genes; and 48 RNAs. The annotation of strain MTB20_M revealed 402 subsystems with 104 virulence, disease, and defense subsystems and detected 4,320 protein-coding genes and 48 RNAs.

The three genomes were compared by performing whole-genome alignment with Mauve (8) to identify sequence differences, and analysis of antimicrobial resistance genes with ResFinder version 2.1 (9) indicated the presence of multiple genes encoding resistance to rifampin (*rpoB*, *rpoC*) and isoniazid. We detected the *katG* gene for MTB1_M and MTB2_M, the *kasA* gene for MTB20_M, and the *inhA* gene for MTB1_M and MTB20_M. For all of the strains we detected second-line resistance to fluoroquinolone.

### Accession number(s).

This whole-genome shotgun project has been deposited at in ENA/GenBank under accession numbers FMDE00000000, FUFR00000000, and FUFS00000000 for *M. tuberculosis* strains MTB2_M, MTB1_M, and MTB20_M, respectively. The version described in this paper for MTB2_M is the third version (FMDE030000000), and the versions for MTB1_M and MTB20_M are the first versions (FUFR01000000 and FUFS01000000, respectively).
